# Screening for Atrial Fibrillation in Stroke Prevention: A Systematic Review and Meta-Analysis of Randomized Controlled Trials

**DOI:** 10.31083/RCM36262

**Published:** 2025-07-23

**Authors:** Xueru Yang, Jun Huang, Ziqian Huang, Yumei Xue, Hai Deng, Xi Cao

**Affiliations:** ^1^The School of Nursing, Sun Yat-Sen University, 510080 Guangzhou, Guangdong, China; ^2^Department of Geriatrics, Guangdong General Hospital, Institute of Geriatrics, Guangdong Provincial People’s Hospital (Guangdong Academy of Medical Sciences), Southern Medical University, 510080 Guangzhou, Guangdong, China; ^3^Guangdong Provincial Key Laboratory of Clinical Pharmacology, Guangdong Cardiovascular Institute, Guangdong Provincial People’s Hospital (Guangdong Academy of Medical Sciences), Southern Medical University, 510080 Guangzhou, Guangdong, China

**Keywords:** atrial fibrillation, screening, stroke, meta-analysis

## Abstract

**Background:**

Evidence is needed to determine the benefits and harms of screening for atrial fibrillation (AF) in stroke prevention. This meta-analysis aimed to evaluate the benefits and issues of AF screening among older adults.

**Methods:**

This systematic review and meta-analysis were conducted and reported in accordance with the Preferred Reporting Items for Systematic Reviews and Meta-Analyses (PRISMA) statement. We systematically searched several databases from inception through 28 March 2025, selecting randomized controlled trials (RCTs) comparing AF screening, including systematic and opportunistic screening, versus routine practice or no screening. Two reviewers independently extracted the data and appraised the risks of bias of the studies.

**Results:**

Thirteen articles covering 12 RCTs were included in the meta-analysis. For routine screening, systematic screening, rather than opportunistic screening, was more effective in detecting new AF cases (relative risk (RR), 2.07; 95% CI, 1.41 to 3.04; *p* = 0.0002). However, no difference was observed in the effectiveness of systematic and opportunistic screening in detecting AF (RR, 1.39; 95% CI, 0.59 to 3.30; *p* = 0.45). Compared with no screening, single-time-point screening did not improve the AF detection rate, whereas intermittent/continuous screening was associated with a greater likelihood of detecting AF (RR, 2.40; 95% CI, 1.59 to 3.64; *p* < 0.0001). There were no significant differences in the anticoagulation prescription rate between patients who underwent screening and routine care (RR, 1.16; 95% CI, 0.94 to 1.44; *p* = 0.16). Systematic screening was associated with a lower risk for the composite endpoint (combination of thrombosis-related events and mortality; RR, 0.96; 95% CI, 0.93 to 0.99; *p* = 0.02) but not for the individual endpoints. Compared with routine care, systematic screening did not increase the risk of major bleeding (RR, 0.88; 95% CI, 0.72 to 1.06; *p* = 0.18), whereas a positive screening result could promote anxiety.

**Conclusions:**

Systematic screening outperformed routine care but was comparable to opportunistic screening in detecting undiagnosed AF. Systematic screening was related to a reduction in the composite endpoints of stroke and all-cause mortality without increasing the risk of bleeding.

**PROSPERO Registration:**

This systematic review was prospectively registered in PROSPERO, registration number: CRD42024558614, https://www.crd.york.ac.uk/PROSPERO/view/CRD42024558614.

## 1. Introduction

Atrial fibrillation (AF) affects approximately 60 million people worldwide, and its incidence is increasing annually [1]. With the aging of the population and the increasing prevalence of chronic risk factors such as hypertension among individuals, the prevalence of AF is expected to continue increasing [1, 2] both in developed [3, 4] and developing countries [5]. AF carries great risks for multiple adverse health outcomes, such as stroke, heart failure, mortality, and disability. Among these outcomes, AF increases the risk of ischemic stroke by 4- to 5-fold [6], and approximately 30% of ischemic strokes are attributed to AF [7]. AF-induced stroke is more devastating than other types of stroke, with higher reported rates of mortality and disability [6, 7]. Effective oral anticoagulation can reduce the risk of stroke once AF is detected. However, a considerable number of AF cases remain undetected due to the asymptomatic and paroxysmal nature of the disease [8, 9, 10], leaving these patients unnoticed, untreated, and at an accordingly elevated risk of stroke.

Screening for AF in high-risk populations, such as adults ≥65 years, is recommended to identify undiagnosed AF early and initiate anticoagulation therapy for preventing stroke [11]. With technological advances and the development of effective anticoagulant treatments, a variety of AF screening initiatives, such as SAFE [12], STROKESTOP [13], AF-CATCH [14], and REHEAR-AF [15], have emerged and shown effectiveness in identifying patients with undiagnosed AF; however, the diagnostic yields of these screening programs vary. Furthermore, systematic reviews on the benefits of screening for AF have reported mixed conclusions on the optimal screening approach. For example, two network meta-analyses reported higher diagnostic yields associated with systematic screening than with opportunistic screening for the elderly population [16, 17], whereas two more recent reviews failed to identify an optimal screening approach [18, 19]. Additionally, widespread interest in the outcomes of these screening programs has been reported in previous systematic reviews, such as Whitfield *et al*.’s review [17] examining the effects of screening on the AF detection rate alone, whereas Bonander *et al*. [19] focused on the effects on stroke without reporting the AF detection rate. Therefore, a consensus on whether screening for AF could improve health outcomes and which approach is the most effective has not yet been reached.

Professional guidelines have made different recommendations regarding AF screening. The European Society of Cardiology [11] recommends opportunistic screening for AF for people ≥65 years of age, and systematic screening could be considered for individuals ≥75 years or those at high risk of stroke. Owing to the limited data available to determine the balance of benefits and harms of AF screening, the United States Preventive Services Task Force [20, 21] indicate that there is insufficient evidence to make a recommendation on screening for AF. Before making the decision to implement or scale up an AF screening initiative, policymakers need assurance that the benefits of the screening outweigh its harms.

With several new published randomized controlled studies (RCTs) (e.g., the GUARD-AF) evaluating the clinical benefits of screening for AF, timely, systematic re-evaluations of the benefits and harms of AF screening are warranted. Therefore, we performed this systematic review with the goal of answering the following research questions:

(1) Is screening more effective than routine care in detecting undiagnosed AF? If so, which screening approach (systematic screening versus opportunistic screening) is more effective?

(2) Do AF patients identified via screening benefit from anticoagulation therapy for preventing stroke?

(3) Does screening for AF result in any adverse events (e.g., major bleeding related to the use of anticoagulants or screening-induced anxiety)?

## 2. Methods

### 2.1 Data Sources and Searches

Five English databases (MEDLINE (https://ovidsp.dc2.ovid.com/ovid-new-b/ovidweb.cgi), EMBASE (https://www.embase.com/search/quick), PubMed, Cochrane library (https://www.cochranelibrary.com/), CINAHL (https://research.ebsco.com/c/2wae6m/search)) and two Chinese databases (China Biology Medicine disc (https://www.sinomed.ac.cn/index.jsp), China National Knowledge Infrastructure (CNKI) (https://www.cnki.net/)) were searched for English- and Chinese-language articles published from database inception to March 28, 2025. The search terms used were as follows: (Atrial fibrillation OR AF OR AFb OR auricular fibrillation* OR atrium fibrillation* OR af OR a-fib OR atrial flutter* OR auricular flutter*) AND (Screening OR screen OR detect* OR identif* OR diagnos* OR test*) AND (randomized controlled trial OR RCT OR controlled trial OR controlled study) (**Supplementary Table 1**). The bibliographies of the included articles were also searched for additional studies. Web searches, such as on Google Scholar, were also performed for relevant studies not retrieved through the database searches. The protocol for this review was prospectively registered with PROSPERO (CRD42024558614). This systematic review was performed in accordance with the Preferred Reporting Items for Systematic Review (PRISMA) guidelines.

### 2.2 Study Selection 

All retrieved records were imported into Covidence for screening. After duplicate studies were removed, two review authors (XRY, ZQH) independently read the titles and abstracts of the articles and retrieved the full texts of the studies that might have met the criteria listed below for further examination.

#### 2.2.1 Types of Studies

All randomized controlled studies were eligible for inclusion. If multiple reports were published on the same study, we treated these reports as one study and pooled the data from those with the longest reported follow-up. We excluded diagnostic studies that focused primarily on the diagnostic accuracy of the screening tools. Conference abstracts, reviews, letters to the editor, or editorials were also excluded.

#### 2.2.2 Types of Participants

We focused on studies on adults aged 65 years or above with no history of AF. Studies that included adults less than 65 years of age, those with implantable pacemakers or defibrillators or those with a previous diagnosis of AF were eligible for inclusion only if these patients were excluded from the final data analyses on the original study outcomes. We excluded studies that screened patients for AF after acute cerebrovascular accidents such as stroke.

#### 2.2.3 Types of Intervention

Studies comparing screening for AF, including systematic screening and opportunistic screening, versus routine practice or no screening were eligible for inclusion. According to the definitions in the literature [11, 22, 23], we defined systematic screening as offering tests for AF to a whole target population, such as adults aged 75 years or above, and opportunistic screening as offering tests for AF in a target population at point-of-care, during consultation for another illness, or during events such as immunization sessions. We included studies that used noninvasive tools to screen for AF. In opportunistic screening contexts, the AF test should not have been mandated for the entire population. The method for detecting AF in the intervention group could be single-step or multistep. We also did not limit the intensity used for screening. We defined routine practice as tests in which diagnoses of AF were made during routine care or in routine consultations with the presentation of symptoms.

#### 2.2.4 Types of Outcome Measure

Primary outcomes

• The detection of new cases of AF. A diagnosis of AF was established with standard 12-lead electrocardiogram (ECG) recording or at least 30 s of single-lead ECG tracing showing a heart rhythm with no discernible repeating P waves and irregular relative risk (RR) intervals that was confirmed by a physician or trained ECG technician or nurse.
• A composite endpoint of ischemic stroke/transient ischemic attack (TIA), systemic embolism, and all-cause mortality.

Secondary outcomes

• Anticoagulant prescription rate
• Adverse events associated with screening, such as major bleeding (defined as bleeding that requires hospitalization for treatment) related to anticoagulation following a diagnosis of AF, and psychological distress associated with screening.

### 2.3 Data Extraction and Risk-of-Bias Assessment

Two review authors independently extracted study data using a pre-piloted data extraction form. We extracted the following data from the included studies: relevant data pertaining to study characteristics (e.g., author, date, study setting, participant characteristics, screening strategy and tools, sample size) and data on outcomes of interest. The data required to assess the risk of bias were also extracted.

Two review authors independently assessed the risk of bias of the included studies with the Cochrane Risk of Bias (RoB) tool [24], which can be used to assesses the quality of an RCT study in terms of the following aspects: random sequence generation (selection bias), allocation concealment (selection bias), blinding of participants and personnel (performance bias), blinding of outcome assessment (detection bias), incomplete outcome data (attrition bias), selective reporting (reporting bias) and other sources of bias. A final rating of low risk, unclear risk or high risk of bias was made on the basis of the comprehensive judgment of the above criteria. Publication bias was assessed visually via generation of a funnel plot and through Egger regression.

Disagreements between the two reviewers during the above procedures were resolved via discussion.

### 2.4 Data Synthesis and Analysis

We summarized the study characteristics with descriptive statistics and quantified between-study heterogeneity with the I^2^ statistic, with values <25%, 25–50%, and >50% indicating low, moderate, and high degrees of heterogeneity, respectively. We performed a meta-analysis of similar studies to estimate the pooled effects of screening using RevMan 5.4.1 (Version 5.4, Review Manager, The Cochrane Collaboration, Oxford, UK); a random effects model was employed if a high degree of heterogeneity (I^2^ values >50%) was identified; otherwise, a fixed effects model was used. We performed separate subgroup analyses on the basis of population and screening characteristics. Sensitivity analysis for the primary outcomes was performed to examine the stability of the results via the one-by-one elimination method in Stata 12.0 (StataCorp, College Station, TX, USA). We generated a funnel plot to visually assess publication bias, and Egger regression was used to evaluate the statistical significance of the bias. We narratively summarized the findings for data that could not be quantitatively pooled. A two-tailed *p* < 0.05 was considered to indicate statistical significance.

## 3. Results

### 3.1 Study Selection and Characteristics of the Included Studies

**Fig. 1.**
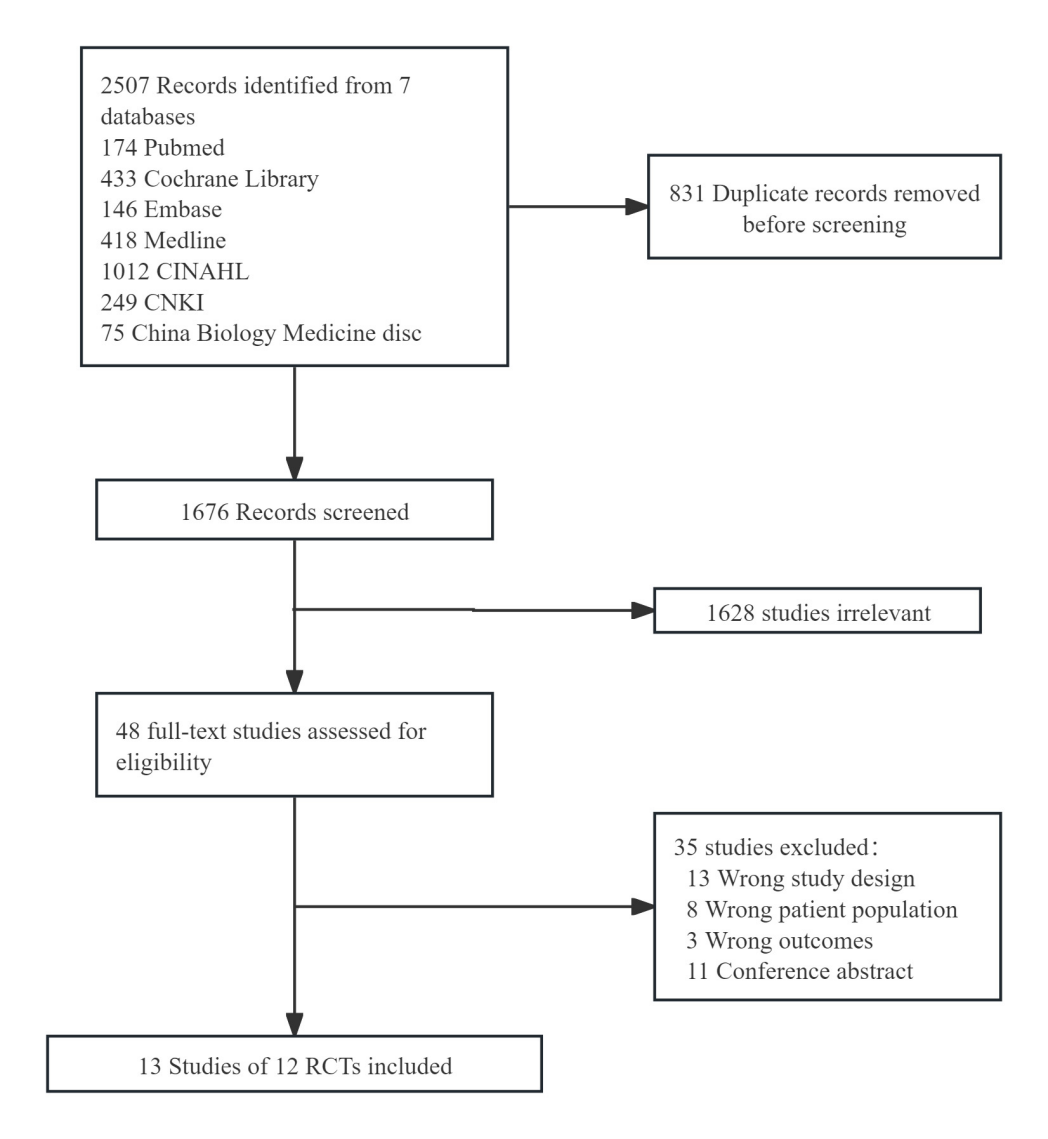
**Study flow diagram**. CNKI, China National Knowledge Infrastructure; RCTs, randomized controlled studies.

Of the 2507 records identified from the initial database search, 1676 records screened for eligibility by a reading of the title and abstract after duplicates were removed. We retrieved the full texts of 48 articles for further assessment and ultimately included 13 articles covering 12 RCTs for meta-analysis [13, 14, 15, 25, 26, 27, 28, 29, 30, 31, 32, 33]. Fig. 1 illustrates the flowchart of the study selection process.

A total of 135,278 patients were involved in these studies, with sample sizes ranging from 200 to 30,715 per study. Among the 12 RCTs, 11 were parallel group trials [13, 14, 15, 25, 27, 28, 29, 30, 31, 32, 33], and one was a multi-arm study [26]. Eight studies [14, 15, 25, 26, 28, 30, 31, 32] included older adults aged ≥65 years with or without additional stroke risk factors, and four studies [13, 27, 29, 33] included patients aged ≥70 or ≥75 years. The screening methods used varied across studies, with handheld or adhesive single-lead ECGs being the most frequently used devices. Seven studies were from European countries, two were from the United States, one was from Australia, one was from China, and one multi-center study was from Canada and Germany. The majority of the studies were conducted in the community. The characteristics of the included studies are depicted in **Supplementary Table 2**.

### 3.2 Risk of Bias Assessment and Level of Evidence

Among the 12 studies, 5 had a low risk of bias [15, 26, 27, 32, 33], whereas the remaining studies had unclear or high risks of bias [13, 14, 25, 28, 29, 30, 31]. Regarding the specific sources of bias, selection bias arising from random sequence generation and allocation concealment, detection bias from outcome assessment blinding, and attrition bias were the main concerns (**Supplementary Figs. 1,2**).

### 3.3 Benefits of Screening for AF

#### 3.3.1 AF Detection Rate

Seven studies [13, 14, 15, 25, 27, 29, 33] compared systematic screening versus routine care, three studies [28, 30, 32] compared opportunistic screening versus routine care, and two studies [26, 31] involved comparisons of systematic screening and opportunistic screening in AF detection.

**Fig. 2.**
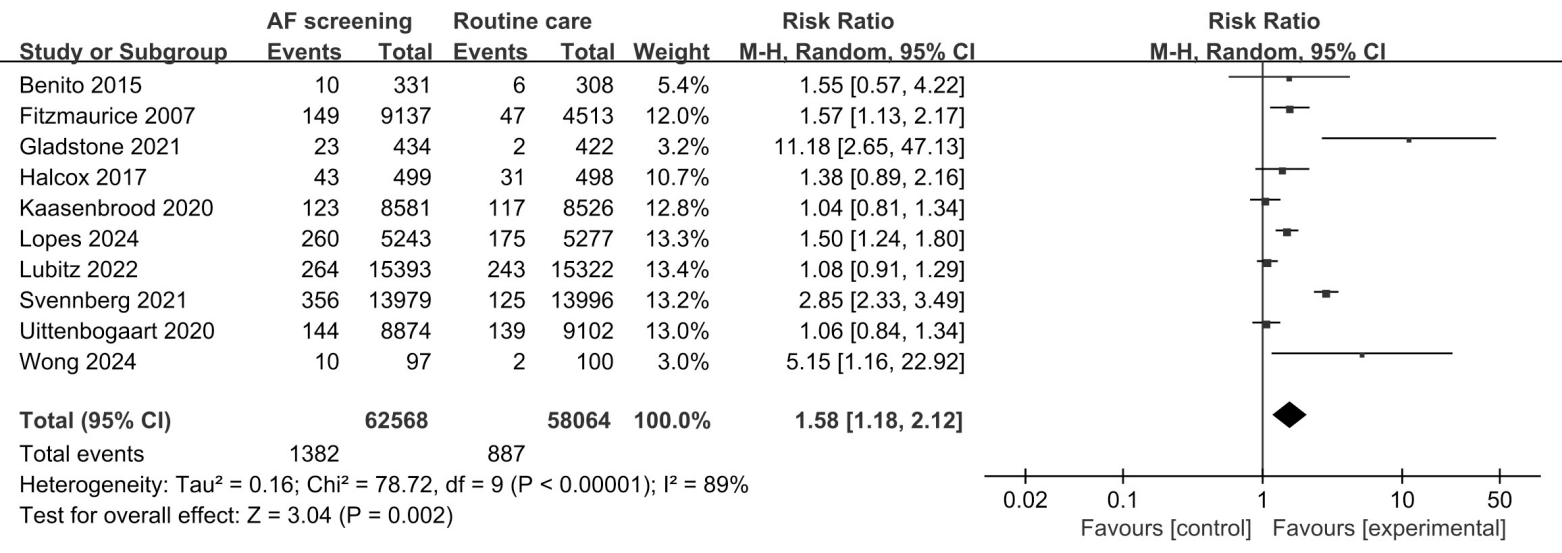
**Detection of new cases of atrial fibrillation: screening versus routine practice**. AF, atrial fibrillation.

**Fig. 3.**
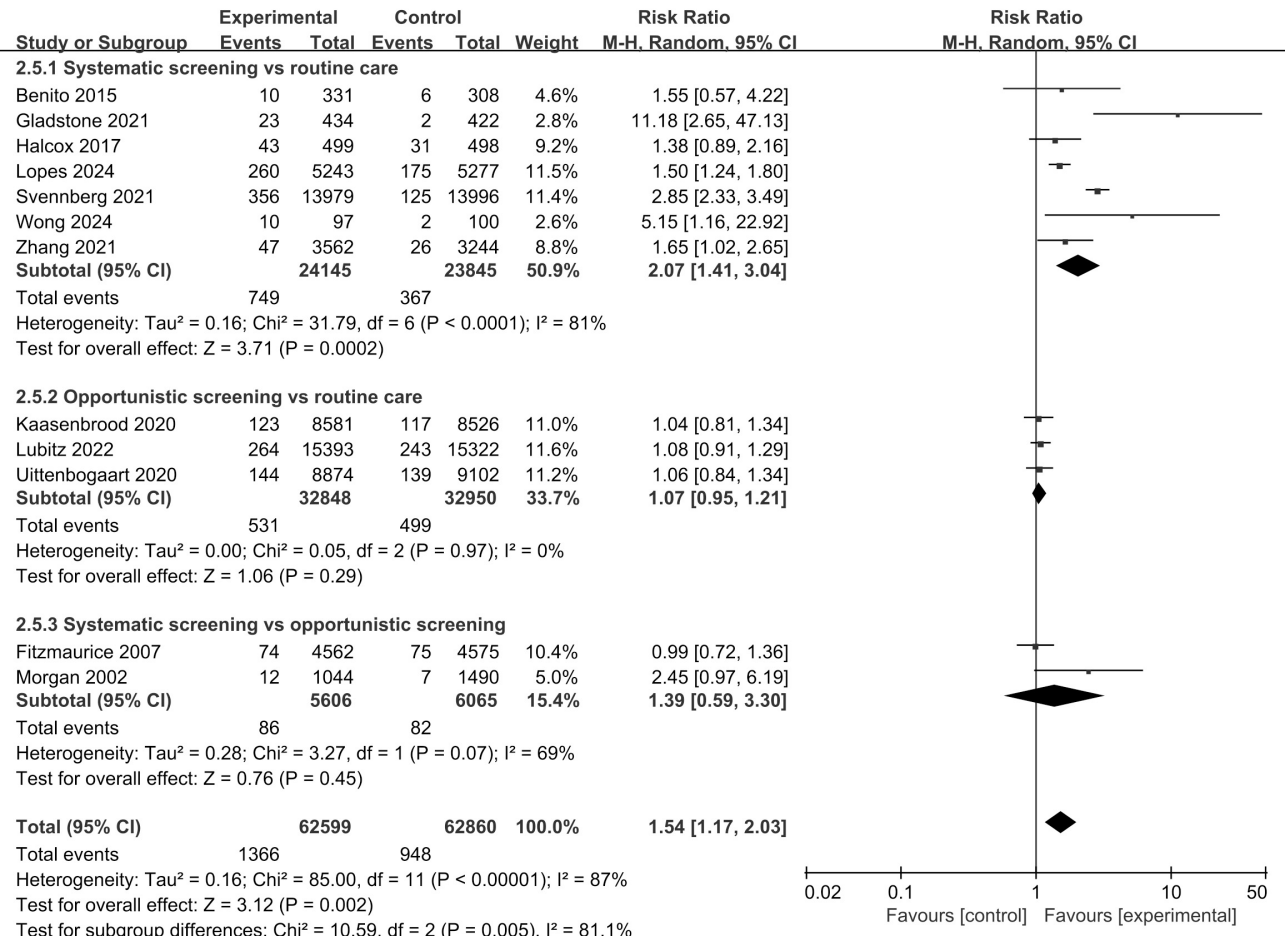
**Detection of new AF cases: subgroup analysis of screening strategies**.

Overall, screening was more effective in detecting new cases of AF than was routine practice (RR, 1.58; 95% CI, 1.18 to 2.12; *p* = 0.002) (Fig. 2). With respect to routine practice, systematic screening and opportunistic screening resulted in the increased detection of new AF cases (RR, 2.07; 95% CI, 1.41 to 3.04; *p* = 0.0002) (Fig. 3). However, we found no difference in the effectiveness of systematic screening versus opportunistic screening for AF detection when data from the two relevant studies were pooled (RR, 1.39; 95% CI, 0.59 to 3.30; *p* = 0.45).

The heterogeneity test showed that I^2^ was 89%, which was greater than 50%, indicating significant heterogeneity among the studies. We performed subgroup analyses according to age and intensity of screening. Compared with routine care, screening of older adults ≥65 or 70 years resulted in increased AF detection (RR, 1.30; 95% CI, 1.07 to 1.58; *p* = 0.010; RR, 2.82; 95% CI, 1.54 to 5.14; *p* = 0.0007), with yields increasing with age (**Supplementary Fig. 3**). Compared with no screening, single-time-point screening did not improve the AF detection rate, whereas intermittent/continuous screening was associated with greater AF yields (**Supplementary Fig. 4**); this difference was the main source of heterogeneity in this meta-analysis.

#### 3.3.2 Thrombosis-Related Events and All-Cause Mortality 

**Fig. 4.**
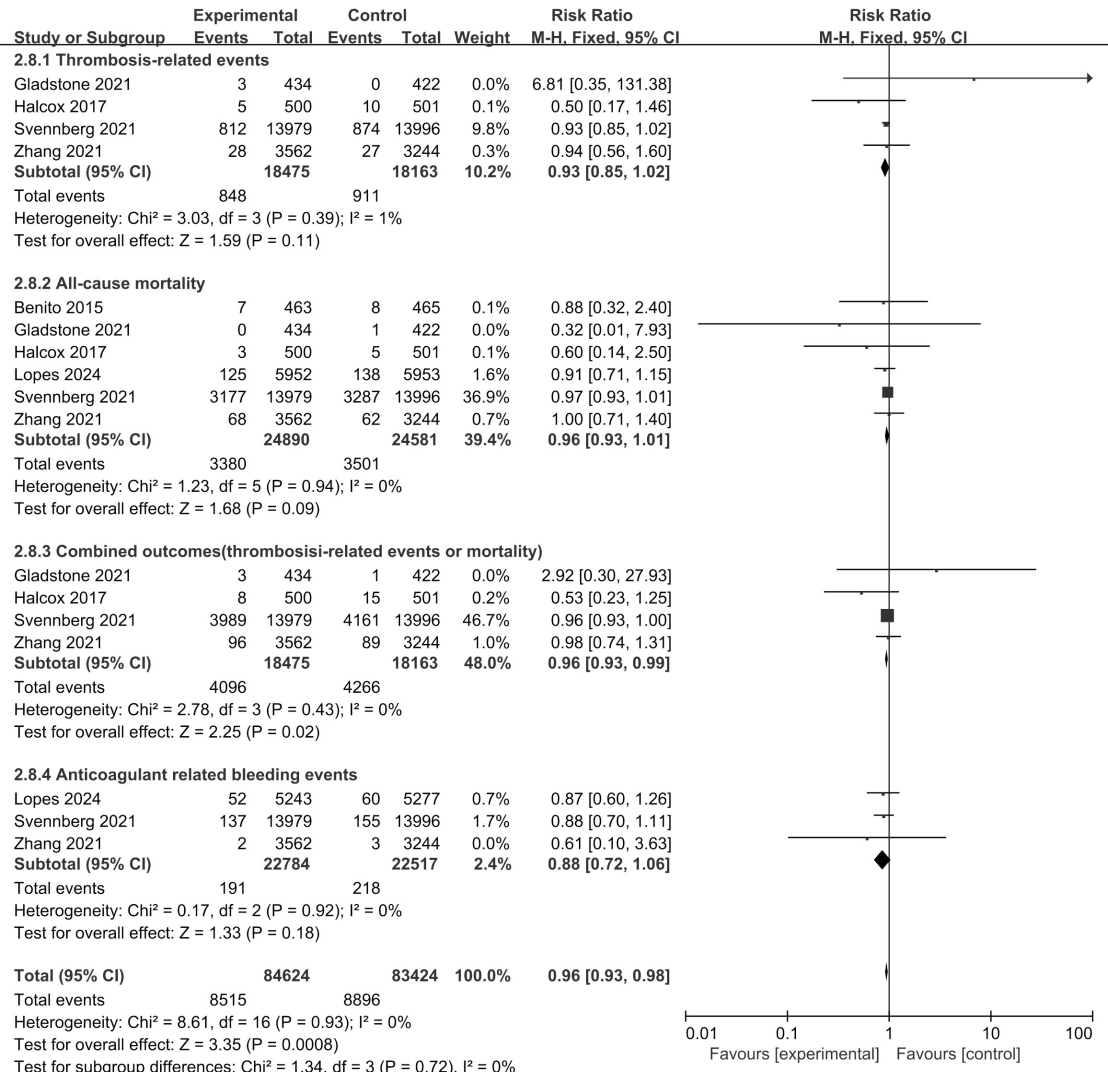
**Benefits and harms of screening for AF for stroke prevention: screening versus routine care**.

Four studies [13, 14, 15, 27] compared systematic screening and routine practice in terms of stroke prevention, and six reported the endpoint of all-cause mortality [13, 14, 15, 25, 27, 29]. Compared with usual care, systematic screening was associated with a lower risk for the composite endpoint (combination of thrombosis-related events and mortality) (RR, 0.96; 95% CI, 0.93 to 0.99; *p* = 0.02) but not for the individual endpoints (Fig. 4).

#### 3.3.3 Anticoagulation Prescription

Seven studies [13, 15, 25, 27, 28, 29, 30] reported the effects of screening on anticoagulation prescriptions. Pooling of the results revealed no significant differences in the anticoagulation prescription rate between patients managed with screening and those managed with routine care (RR, 1.16; 95% CI, 0.94 to 1.44; *p* = 0.16) (**Supplementary Fig. 5**).

### 3.4 Adverse Events Associated With AF Screening

Three studies reported bleeding events, including hemorrhagic stroke and other major bleeding associated with anticoagulation [13, 14, 29]. These studies all compared systematic screening with routine care; synthesis of these studies revealed that systematic screening was not associated with a greater risk of major bleeding than routine care (RR, 0.88; 95% CI, 0.72 to 1.06; *p* = 0.18) (Fig. 4). One study [12] reported no significant differences in anxiety or quality of life between the screened group and the non-screened group, but patients with positive screening results experienced more anxiety and lower quality of life than those with negative screening results. In the REHEARSE-AF study, participants in the screening group were more aware of their AF risk but were not very anxious about the illness [15].

### 3.5 Sensitivity Analysis and Publication Bias

We performed sensitivity analysis by excluding individual studies one by one and found that the intervention effects on the AF detection rate were stable. However, caution should be taken when generalizing the results of the effectiveness of screening for AF on the composite endpoint and major bleeding due to the instability of these results as indicated by the sensitivity analysis. We found no substantial publication bias in this review on the basis of the results of Egger regression and funnel plot analysis (**Supplementary Figs. 6,7**).

## 4. Discussion

This systematic review examined the benefits of screening for AF in older adults. We found that compared with routine care, systematic screening, in addition to opportunistic screening, resulted in a significant increase in the detection of AF. We did not find a significant difference in AF detection between systematic screening and opportunistic screening however. Compared with usual care, systematic screening was associated with a lower risk of the composite endpoint (the combination of thrombosis-related events and all-cause death) without increasing the risk of bleeding. However, systematic screening did not improve the anticoagulation prescription rate, and screening positivity might result in anxiety.

Conflicts exist in the literature regarding the effectiveness of AF detection methods. Two meta-analyses [16, 17] of 9 studies (SCREEN-AF, STROKESTOP, D2AF, SAFE, EARLY, mSTOPS, HECTOR-AF, Kaasenbroad and Morgan studies) involving more than 80,000 patients indicated that systematic screening, as well as opportunistic screening, was associated with a higher AF detection rate in people aged 65 years or above than no screening was. The results of our review agree with those of these two reviews on the effectiveness of systematic screening and the lack of significant benefits with opportunistic screening for detecting AF in individuals aged 65 years or older. When comparing systematic versus opportunistic screening, our review revealed comparable efficacy between the two methods; conversely, the above two reviews suggested that systematic screening was superior to opportunistic screening [16, 17]. Our review (n = 12) included more RCTs than the above two meta-analysis studies did, however, and therefore may provide a more reliable estimation of the true effects of intervention screening over usual care. In the current analysis, we limited screening tools to noninvasive devices, including 12-lead ECGs, single-lead ECGs and photoplethysmography (PPG) devices. Despite the reported high sensitivity of these tools in detecting AF, differences in specificity remain, which may result in false positive results and thus affect AF yields [34]. Currently, there is no consensus on which screening strategy (systematic versus opportunistic screening) is more effective in detecting AF. The results of the current and previous reviews on systematic and opportunistic screening were driven mainly by the SAFE study [12, 26] and the Morgan [31] study, which compared single-time point systematic screening with 12-lead ECG versus opportunistic pulse-taking with confirmatory ECG but yielded mixed results. Thus, findings from these reviews should be interpreted with caution due to potential bias in the included studies and the imprecision in the results, which weakened the overall findings.

Despite the yields in AF, the cost-effectiveness of the screening program is an important concern in the implementation or scaling up of the program for policy makers. In a meta-analysis [35] of 5 studies, opportunistic screening was more likely to be cost-effective than a systematic approach was; however, the meta-analysis failed to consider the impact of screening intensity. In general, intensive screening results in high diagnostic yield but also high costs, whereas less intensive screening results in low costs but low detection rates. Our review demonstrated that intermittent and continuous screening was associated with increased AF yields; in contrast, single-time-point screening did not increase AF yields over routine care. Similar results were also reported in the study by Kaasenbrood *et al*. [28]. However, we failed to compare approaches with different intensities from the perspective of cost-effectiveness. Previous cost-effectiveness studies have suggested that systematic screening is more cost effective than no screening for preventing stroke in patients ≥65 years of age [36, 37], but direct evidence on the optimal intensity of screening remains lacking. Future economic studies are warranted to explore this topic.

Controversies also exist regarding AF screening among professional guidelines. For example, in the European Society of Cardiology [11] and Chinese Society of Cardiology [38] guidelines, opportunistic screening for AF detection and systematic screening should be considered according to the risk of stroke of the individual, whereas Canadian [39] and Austrian [22] guidelines recommend opportunistic, point-of-care screening but make no recommendations for systematic screening. The United States Preventive Services Task Force [20] emphasizes the lack of sufficient evidence to make a recommendation on screening for AF.

The goal of screening for AF is to initiate timely treatment and reduce the AF burden and stroke risk. However, we found no significant difference in the risk for the individual endpoints of thrombosis-related events and all-cause mortality between screening and usual care. Consistent with our results, Elbadawi *et al*. [16] reported no significant differences in all-cause mortality or cardiovascular accidents among various AF screening approaches in a synthesis of 9 studies on noninvasive tools for detecting AF in elderly individuals. Caution should be taken when interpreting these results due to the limited number of stroke events in each individual study, which may limit the power to estimate the true intervention effects. In terms of the risk for the composite endpoint, we found significant improvements with systematic screening. The decreased risk of the composite event might have resulted from increased visits during systemic screening and referrals for or the resolution of health issues aside from AF [40]. Unlike Elbadawi *et al*.’s review [16], which reported higher rates of oral anticoagulant (OAC) use with systematic screening, we did not find significant improvements in OAC prescription rates with screening, which may explain the nonsignificant decrease in thrombosis-related events described above. Screening for AF might result in adverse events, such as bleeding related to anticoagulant use and anxiety induced by false positive results. Our findings revealed no increased risk of major bleeding associated with systematic screening over usual care. The SAFE study [12, 26] reported anxiety and impaired quality of life related to positive screening results, but more evidence is needed to determine the clinical relevance of these changes.

## 5. Limitations

This review has several limitations. First, a high level of heterogeneity was observed among the included studies on the AF detection rate, although subgroup analysis was performed to explore the causes of the heterogeneity. Second, the limited number of clinical outcomes (e.g., stroke, death, major bleeding) reported in some of the trials may decrease the power of this review in detecting the true effects of screening. Third, the variability in participants might weaken the generalizability of the findings.

## 6. Conclusions 

The burden of AF continues to rise, especially in older adults. Screening for undiagnosed AF allows early diagnosis and initiation of treatment, reducing the burden of AF and the risk of stroke. However, additional evidence is needed to determine the benefits and harms of screening for AF. The current systematic reviews included 12 RCTs involving 135,278 patients and consolidated the role of screening over usual care in improving AF yields, driven mainly by systematic screening approaches. We found no difference in the AF detection rate between systematic screening and opportunistic screening. With respect to clinical outcomes, systematic screening was associated with a reduction in the composite endpoint of stroke and all-cause mortality (but not in the individual endpoints) without increasing the risk of bleeding. Caution should be taken when interpreting these findings because of the heterogeneity among and potential bias in the included studies.

## Availability of Data and Materials

The datasets used and analyzed during the current study are available from the corresponding author on reasonable request.

## Supplementary Material

Supplementary material associated with this article can be found, in the online version, at https://doi.org/10.31083/RCM36262.
