# Supplementary material for: Screening for Atrial Fibrillation in Stroke Prevention: A Systematic Review and Meta-Analysis of Randomized Controlled Trials
Source: Rev Cardiovasc Med. 2025 Jul 23;26(7):36262. doi: 10.31083/RCM36262 (PMC12326414; doi:10.31083/RCM36262)
Supplement: Supplementary file 1 [file 2153-8174-26-7-36262-s1.zip › Supplementary Fig 1.pdf]

	Random sequence generation (selection bias)	Allocation concealment (selection bias)	Blinding of participants and personnel (performance bias)	Blinding of outcome assessment (detection bias)	Incomplete outcome data (attrition bias)	Selective reporting (reporting bias)	Other bias
Benito 2015	?	+	?	?	−	+	+
Fitzmaurice 2007	+	+	+	+	+	+	+
Gladstone 2021	+	+	+	+	+	+	+
Halcox 2017	+	+	+	+	+	+	+
Kaasenbrood 2020	+	+	+	?	+	+	+
Lopes 2024	+	+	+	−	+	+	+
Lubitz 2022	?	?	+	+	+	+	+
Morgan 2002	+	+	?	−	−	+	+
Svennberg 2021	+	+	+	−	+	+	+
Uittenbogaart 2020	+	+	+	+	+	+	+
Wong 2024	+	+	+	+	+	+	+
Zhang 2021	+	+	+	+	?	+	+
