# Supplementary material for: Screening for Atrial Fibrillation in Stroke Prevention: A Systematic Review and Meta-Analysis of Randomized Controlled Trials
Source: Rev Cardiovasc Med. 2025 Jul 23;26(7):36262. doi: 10.31083/RCM36262 (PMC12326414; doi:10.31083/RCM36262)
Supplement: Supplementary file 1 [file 2153-8174-26-7-36262-s1.zip › Supplementary Material.docx]

**Supplementary**

Table 1. Detailed search strategy **1**

Table 2. Characteristics of the included studies **3**

Fig. 1 Risk of bias using the RoB 1 tool**6**

Fig. 2 Risk percentage for each domain and overall risk of bias using the RoB 1 tool**7**

Fig. 3 Subgroup analysis of AF detection according to age**8**

Fig. 4 Subgroup analysis of AF detection according to intensity of screening**8**

Fig. 5 Meta-analysis on prescription of anticoagulant**9**

Fig. 6 Funnel plot**9**

Fig. 7 Egger’ s test**9**

Supplementary Table 1. Detailed search strategy.

| Cochrane | | |
| --- | --- | --- |
|  | #1 | MeSH descriptor:[Atrial Fibrillation] explode all trees |
|  | #2 | MeSH descriptor:[Mass Screening] explode all trees |
|  | #3 | (Atrial Fibrillation OR Auricular Fibrillation OR Atrium fibrillation OR AF OR a-fib OR Atrial flutter OR Auricular flutter OR atrium fibrillation):ti,ab,kw |
|  | #4 | (Screening OR screen):ti,ab,kw |
|  | #5 | #1 or #3 |
|  | #6 | #2 or #4 |
|  | #7 | #5 and #6 |
|  | #8 | (older OR elder OR elderly OR aged):ti,ab,kw |
|  | #9 | #7 and #8 |
| Embase | | |
|  | #1 | 'atrial fibrillation':ti,ab,kw OR 'auricular fibrillation':ti,ab,kw OR 'af':ti,ab,kw OR 'a-fib':ti,ab,kw OR 'atrial flutter':ti,ab,kw OR 'auricular flutter':ti,ab,kw OR 'atrium fibrillation':ti,ab,kw |
|  | #2 | screening:ti,ab,kw OR screen:ti,ab,kw |
|  | #3 | older:ti,ab,kw OR elder:ti,ab,kw OR elderly:ti,ab,kw OR aged:ti,ab,kw |
|  | #4 | #1 AND #2 AND #3 AND ([controlled clinical trial]/lim OR [randomized controlled trial]/lim) |
| Pubmed | | |
|  | #1 | "Atrial Fibrillation"[Title/Abstract] OR "auricular fibrillation"[Title/Abstract] OR "atrium fibrillation"[Title/Abstract] OR "AF"[Title/Abstract] OR "a-fib"[Title/Abstract] OR "atrial flutter"[Title/Abstract] OR "auricular flutter"[Title/Abstract] OR "atrium fibrillation"[Title/Abstract] |
|  | #2 | "Atrial Fibrillation"[MeSH Terms] |
|  | #3 | "Mass Screening"[MeSH Terms] |
|  | #4 | "screen"[Title/Abstract] OR "screening"[Title/Abstract] |
|  | #5 | #1 OR #2 |
|  | #6 | #3 OR #4 |
|  | #7 | #5 AND #6 AND (clinical trial[Filter] OR randomized controlled trial[Filter]) |
| MEDLINE | | |
|  | #1 | (screening or screen).ab,ti. |
|  | #2 | Mass Screening/ |
|  | #3 | (Atrial Fibrillation or Auricular Fibrillation or Atrium fibrillation or AF or a-fib or Atrial flutter* or Auricular flutter* or atrium fibrillation).ab,ti. |
|  | #4 | Atrial fibrillation/ |
|  | #5 | #1 OR #2 |
|  | #6 | #3 OR #4 |
|  | #7 | #5 AND #6 |
|  | #8 | Limit 7 to (clinical study or clinical trial, all or clinical trial or comparative study or multicenter study or randomized controlled trial) |
| CINAHL | | |
|  | #1 | TI ("screen" OR "screening") OR AB ("screen" OR "screening") |
|  | #2 | TI ("Atrial Fibrillation" OR "Auricular Fibrillation" OR "Atrium fibrillation" OR "AF" OR "a-fib" OR "Atrial flutter" OR "Auricular flutter" OR "atrium fibrillation") OR AB ("Atrial Fibrillation" OR "Auricular Fibrillation" OR "Atrium fibrillation" OR "AF" OR "a-fib" OR "Atrial flutter" OR "Auricular flutter" OR "atrium fibrillation") |
|  | #3 | TI ("older" OR "elder" OR "elderly" OR "aged") OR AB ("older" OR "elder" OR "elderly" OR "aged") |
|  | #4 | #1 AND #2 AND #3 |

MeSH, Medical Subject Headings; AF, atrial fibrillation.

Supplementary Table 2. Characteristics of the included studies.

| Study | Region | Design | Setting | Eligibility of participants | Participants (Intervention group/Control group) | Screening implementer | Intervention | Comparator | Outcomes | Number needed to treat (NNT) | Follow-up |
| --- | --- | --- | --- | --- | --- | --- | --- | --- | --- | --- | --- |
| Morgan *et al.* 2002 [31] | England | RCT | Home | Patients between the ages of 65 and 100 without known AF | 1,499/1,502 | Nurses | Systematic nurse-led screening by pulse palpation and confirmed by electrocardiogram (ECG) | Prompted Opportunistic screening: notes were flagged for pulse check and, if suspicious of AF then a confirmatory ECG | AF detection rate | 93.5 | 6 months |
| Hobbs *et al.* 2005 [12]; Fitzmaurice *et al.* 2007 [26]  (SAFE) | England | Cluster RCT | Primary healthcare centre | Patients ≥65 years without known AF | 9,866 (4,933 for Opportunistic Screening and Systematic Screeing respectively)/4,936 | Nurses | Single-time point systematic screening with 12-lead ECG device;  Opportunistic screening with pulse-taking and 12-lead ECG device if irregular pulse detected | Routine care | Primary outcomes: incidence of new cases of AF detected; incremental cost per case detected;  Secondary outcomes: cost-effectiveness of screening for AF, the methods of ECG interpretation; patients acceptability to AF screening, anxiety, and quality of life relating to AF screening. | 169.5 | 1 year |
| Benito *et al.* 2015 [25]  (EARLY) | Spain | RCT | Primary healthcare centre | With one or more risk of: ≥65 years, hypertension, ischemic heart disease, valvular heart disease, diabetes, and/or congestive heart failure, but without known AF | Randomization: 2,000/2,000 (Included: 463/465) | Doctors lead screening, nurses participate | Systematic screening using ECG every 6 months and self-pulse check once a month over 2 years | Routine care (No screening) | Primary outcomes: incidence of new cases of AF at 6-month;  Secondary outcomes: no. of AF diagnosed during 2-year; complications related to AF or its treatment, origin and means of the initial AF diagnosis | 20.7 | 2 years |
| Halcox *et al.* 2017 [15]  (REHEARSE-AF) | England | RCT | Home | Patients ≥65 years with a CHADS-VASc ≥2, without known AF | 500/501 | Doctors | Systematic screening using single-lead ECG device (AliveCor Kardia monitor) twice weekly over 12 months | Routine care | Primary outcomes: time to diagnosis of AF,  Secondary outcomes: clinical events including stroke or TIA or systemic embolism; death, major bleeding; participants’ experience about screening, intervention cost per AF diagnosis | 41.7 | 4.2 years |
| Kaasenbrood *et al.* 2020 [28] | Netherlands | Cluster RCT | Primary healthcare centre | Patients ≥65 years without known AF | Randomization: 9,542/9,374  (People without atrial fibrillation: 8,581/8,526) | Doctors | Single-time point opportunistic screening with handheld single-lead ECG (MyDiagnostick) | Routine care (no screening) | Primary outcomes: incidence of new AF, anticoagulant rate | 769.2 | 1 year |
| Uittenbogaart *et al.* 2020 [32]  (D_2_AF) | Netherlands | Cluster RCT | Primary healthcare centre | Patients ≥65 years without known AF | 9,400/9,789 | Doctors and nurses | Opportunistic screening using multiple methods including pulse palpation, blood pressure monitor (WatchBP-Home A), handheld Single-lead ECG (MyDiagnostick), and 12-lead ECG if negative of all above three tests, and holter-ECG if negative of 12-lead ECG. | Routine care | Primary outcome: Number of new cases of AF | 147.1 | 1 year |
| Gladstone *et al.*2021 [27]  (SCREEN-AF) | Canada and Germany | Multicentre RCT | Home | Patients ≥75 years with hypertension but without known AF | 434/422 | Doctors | Systematic screening using Zio patch for 2 weeks, one at baseline and another at 3-month, and with blood pressure monitor (WatchBP-Home A, Microlife Corp) recording twice daily for 6-month. | Routine care with pulse palpation and cardiac auscultation at baseline and 6 months | Primary outcomes: detection r of AF,  Secondary outcomes: anticoagulant rate, device adherence, clinical outcomes (e.g., death, ischemic strokes and other systemic embolism, major bleeding | 126.6 | 6 months |
| Svennberg *et al.* 2021 [13]  (STROKESTOP) | Sweden | Multicentre, parallel group, unmasked, RCT | Primary healthcare centre | Residents aged 75 or 76 | 14,387/14,381 | No information | Systematic screening using handheld single-lead ECG (Zenicor II); if no atrial fibrillation with the index ECG, then twice daily for two weeks | Routine care | Primary outcomes: combined endpoint of ischemic or hemorrhagic stroke, systemic embolism, bleeding leading to hospitalization, all-cause mortality;  Secondary outcomes: detection of AF, death from cardiovascular disease (CVD), hospitalization due to CVD, ischemic stroke, a composite of ischemic stroke and systemic thromboembolism, initiation of oral anticoagulant therapy, cost-efficacy analysis. | 27.3 | 5 years |
| Zhang *et al.* 2021 [14]  (AF-CATCH) | China | RCT | Primary healthcare centre | Patients ≥65 years without known AF | 3,562 (2,841 for quarterly screening and 721 for quarterly plus)/3,244 | No information | Systematic screening at quarterly or quarterly plus (screening once per week for the first month in addition to quarterly screening) for 2-year using handheld Single-lead ECG (AliveCor) | Annual screening (Systematic screening once a year) | Primary outcomes: detection of new cases of AF, secondary outcomes: clinical events including all-cause mortality, cardiovascular deaths, hemorrhagic and ischemic stroke, acute coronary syndrome | 60.2 | 2 years |
| Lubitz *et al.* 2022 [30]  (VITAL-AF) | United States | Cluster RCT | Primary healthcare centre | Patients ≥65 years without known AF | 15,393/15,322 | Doctors and assistant | Point-of-care screening at a single time point using handheld single-lead ECG (AliveCor KardiaMobile ECG device) | Routine care (no screening) | Primary outcomes: incidence of newly diagnosed AF,  Secondary outcomes: change in the incidence proportion of AF before and after screening, anticoagulant rate | 1111.1 | 1 year |
| Lopes *et al.* 2024 [29] | United States | RCT | Primary healthcare centre | Patients ≥70 years without known AF | 5,952/5,953 | No information | Systematic screening using Zio patch for 2 weeks | Routine care (no screening) | Primary outcomes: hospitalization for all-cause stroke (ischemic and hemorrhagic), hospitalization for bleeding,  Secondary outcomes: Newly Diagnosed AF, Oral anticoagulation | 61 | 15.3 months |
| Wong *et al.* 2024 [33] | Australia | RCT | Home | Patients ≥75 years without known AF | 100/100 | No information | Self-recorded their ECGs using AliveCor KardiaMobile ECG device once daily on weekdays | Routine care with their general practitioners (GPs) in the first 6 months | Primary outcomes: new AF diagnosis,  Secondary outcomes: Oral anticoagulation, healthcare visits | 12 | 6 months |

Fig. 1 Risk of bias using the RoB 1 tool


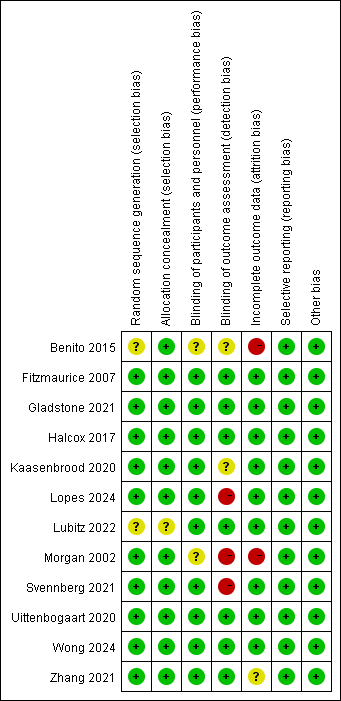


Fig. 2 Risk percentage for each domain and overall risk of bias using the RoB 1 tool


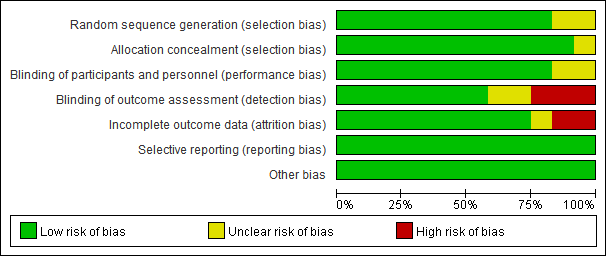


Fig. 3 Subgroup analysis of AF detection according to age


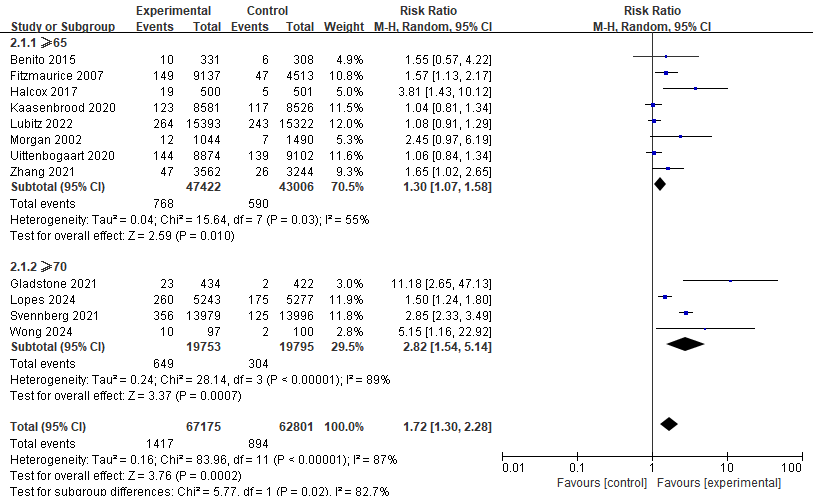


Fig. 4 Subgroup analysis of AF detection according to intensity of screening


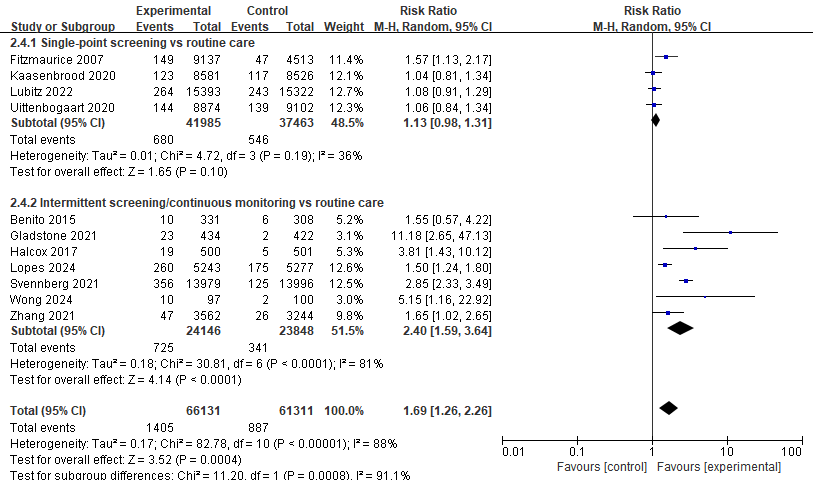


Fig. 5 Meta-analysis on prescription of anticoagulant


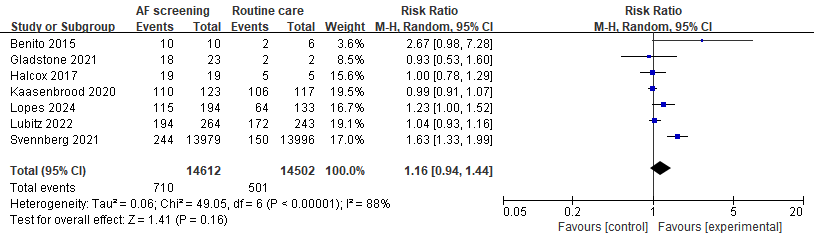


Fig. 6 Funnel plot

Fig. 7 Egger’ s test

**
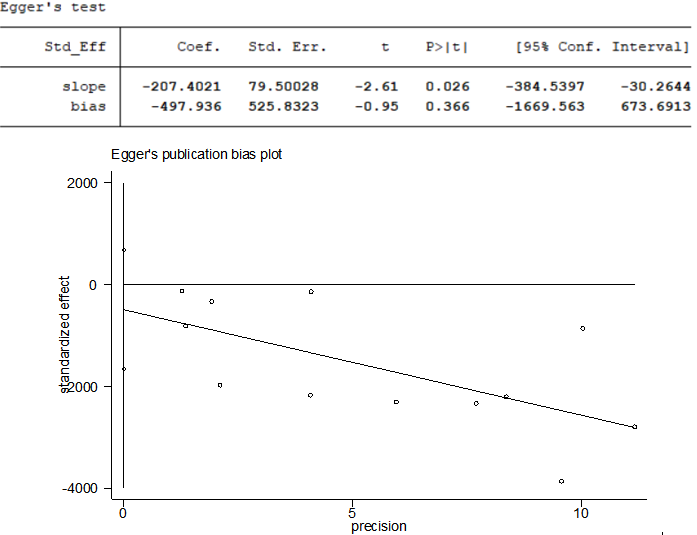
**

**Reference**

[1] Morgan S, Mant D. Randomised trial of two approaches to screening for atrial fibrillation in UK general practice. British Journal of General Practice. 2002; 52: 373-+.

[2] Hobbs FDR, Fitzmaurice DA, Mant J, Murray E, Jowett S, Bryan S, et al. A randomised controlled trial and cost-effectiveness study of systematic screening (targeted and total population screening) versus routine practice for the detection of atrial fibrillation in people aged 65 and over. The SAFE study. Health Technology Assessment. 2005; 9: 1-+. <https://doi.org/DOI> 10.3310/hta9400

[3] Fitzmaurice DA, Hobbs FDR, Jowett S, Mant J, Murray ET, Holder R, et al. Screening versus routine practice in detection of atrial fibrillation in patients aged 65 or over: cluster randomised controlled trial. BMJ. 2007; 335. <https://doi.org/10.1136/bmj.39280.660567.55>

[4] Benito L, Coll-Vinent B, Gomez E, Marti D, Mitjavila J, Torres F, et al. EARLY: a pilot study on early diagnosis of atrial fibrillation in a primary healthcare centre. Europace. 2015. <https://doi.org/10.1093/europace/euv146>

[5] Halcox JPJ, Wareham K, Cardew A, Gilmore M, Barry JP, Phillips C, Gravenor MB. Assessment of Remote Heart Rhythm Sampling Using the AliveCor Heart Monitor to Screen for Atrial Fibrillation: The REHEARSE-AF Study. Circulation. 2017; 136: 1784-1794. <https://doi.org/10.1161/Circulationaha.117.030583>

[6] Kaasenbrood F, Hollander M, de Bruijn SHM, Dolmans CPE, Tieleman RG, Hoes AW, Rutten FH. Opportunistic screening versus usual care for diagnosing atrial fibrillation in general practice: a cluster randomised controlled trial. British Journal of General Practice. 2020; 70: e427-e433. <https://doi.org/10.3399/bjgp20X708161>

[7] Uittenbogaart SB, Verbiest-van Gurp N, Lucassen WAM, Winkens B, Nielen M, Erkens PMG, et al. Opportunistic screening versus usual care for detection of atrial fibrillation in primary care: cluster randomised controlled trial. BMJ. 2020. <https://doi.org/10.1136/bmj.m3208>

[8] Gladstone DJ, Wachter R, Schmalstieg-Bahr K, Quinn FR, Hummers E, Ivers N, et al. Screening for Atrial Fibrillation in the Older Population. JAMA Cardiology. 2021; 6. <https://doi.org/10.1001/jamacardio.2021.0038>

[9] Svennberg E, Friberg L, Frykman V, Al-Khalili F, Engdahl J, Rosenqvist M. Clinical outcomes in systematic screening for atrial fibrillation (STROKESTOP): a multicentre, parallel group, unmasked, randomised controlled trial. Lancet. 2021; 398: 1498-1506. <https://doi.org/10.1016/S0140-6736(21)01637-8>

[10] Zhang W, Chen Y, Miao C-Y, Huang Q-F, Sheng C-S, Shao S, et al. Quarterly versus annual ECG screening for atrial fibrillation in older Chinese individuals (AF-CATCH): a prospective, randomised controlled trial. The Lancet Healthy Longevity. 2021; 2: e470-e478. <https://doi.org/10.1016/s2666-7568(21)00138-0>

[11] Lubitz SA, Atlas SJ, Ashburner JM, Lipsanopoulos ATT, Borowsky LH, Guan W, et al. Screening for Atrial Fibrillation in Older Adults at Primary Care Visits: VITAL-AF Randomized Controlled Trial. Circulation. 2022; 145: 946-954. <https://doi.org/10.1161/circulationaha.121.057014>

[12] Lopes RD, Atlas SJ, Go AS, Lubitz SA, McManus DD, Dolor RJ, et al. Effect of Screening for Undiagnosed Atrial Fibrillation on Stroke Prevention. Journal of the American College of Cardiology. 2024; 84: 2073-2084. <https://doi.org/10.1016/j.jacc.2024.08.019>

[13] Wong KC, Nguyen TN, Marschner S, Turnbull S, Indrawansa AB, White R, et al. A randomised controlled implementation study integrating patient self-screening with a remote central monitoring system to screen community-dwellers aged 75 years and older for atrial fibrillation. European Journal of Preventive Cardiology. 2024: zwae312. <https://doi.org/10.1093/eurjpc/zwae312>
