# Supplementary figures and images for: Screening for Atrial Fibrillation in Stroke Prevention: A Systematic Review and Meta-Analysis of Randomized Controlled Trials
Source: Rev Cardiovasc Med. 2025 Jul 23;26(7):36262. doi: 10.31083/RCM36262 (PMC12326414; doi:10.31083/RCM36262)

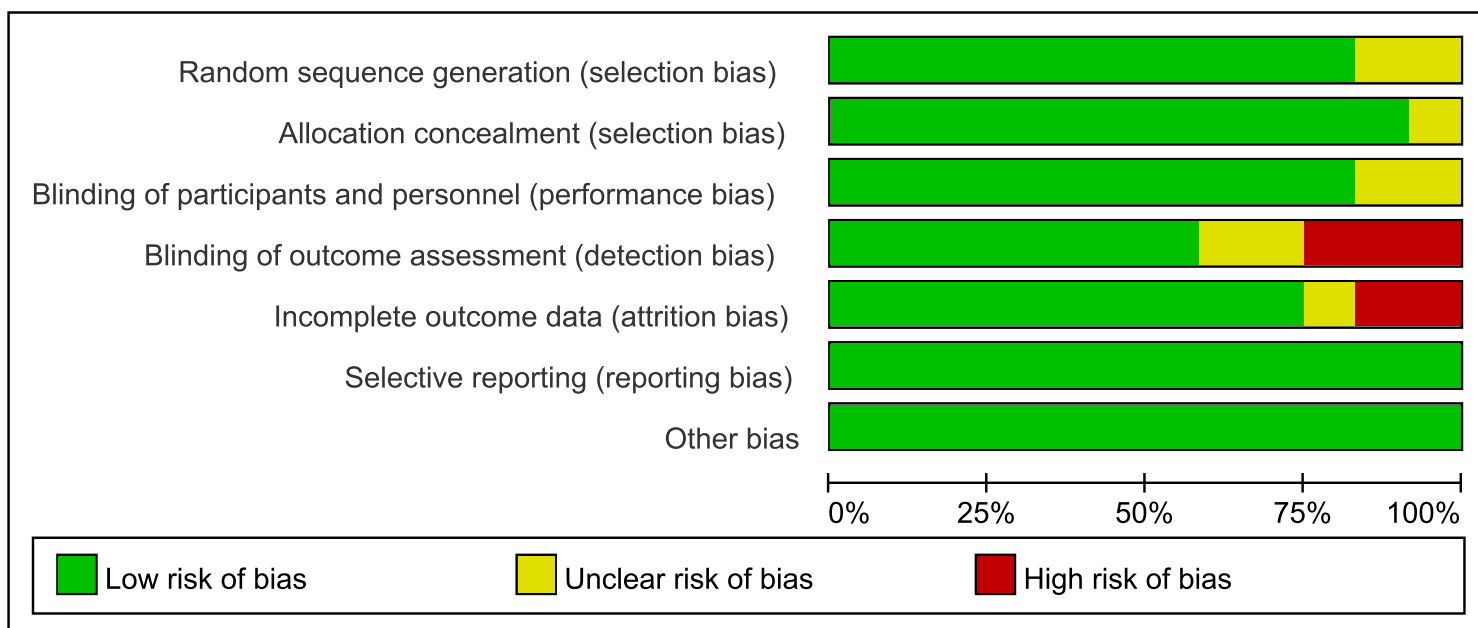

Supplement: Supplementary file 1 [file 2153-8174-26-7-36262-s1.zip › Supplementary Fig 2.pdf]

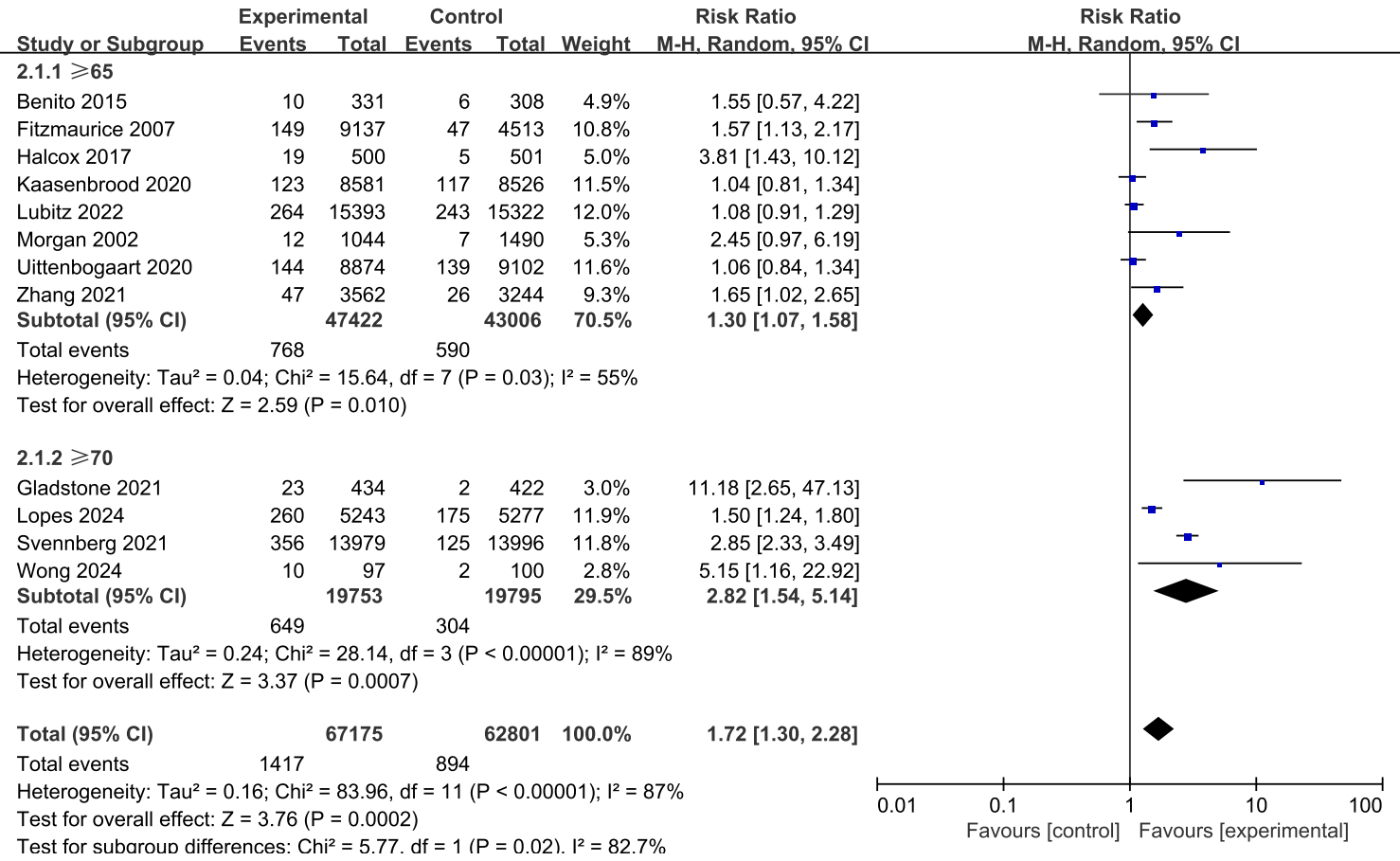

Supplement: Supplementary file 1 [file 2153-8174-26-7-36262-s1.zip › Supplementary Fig 3.pdf]

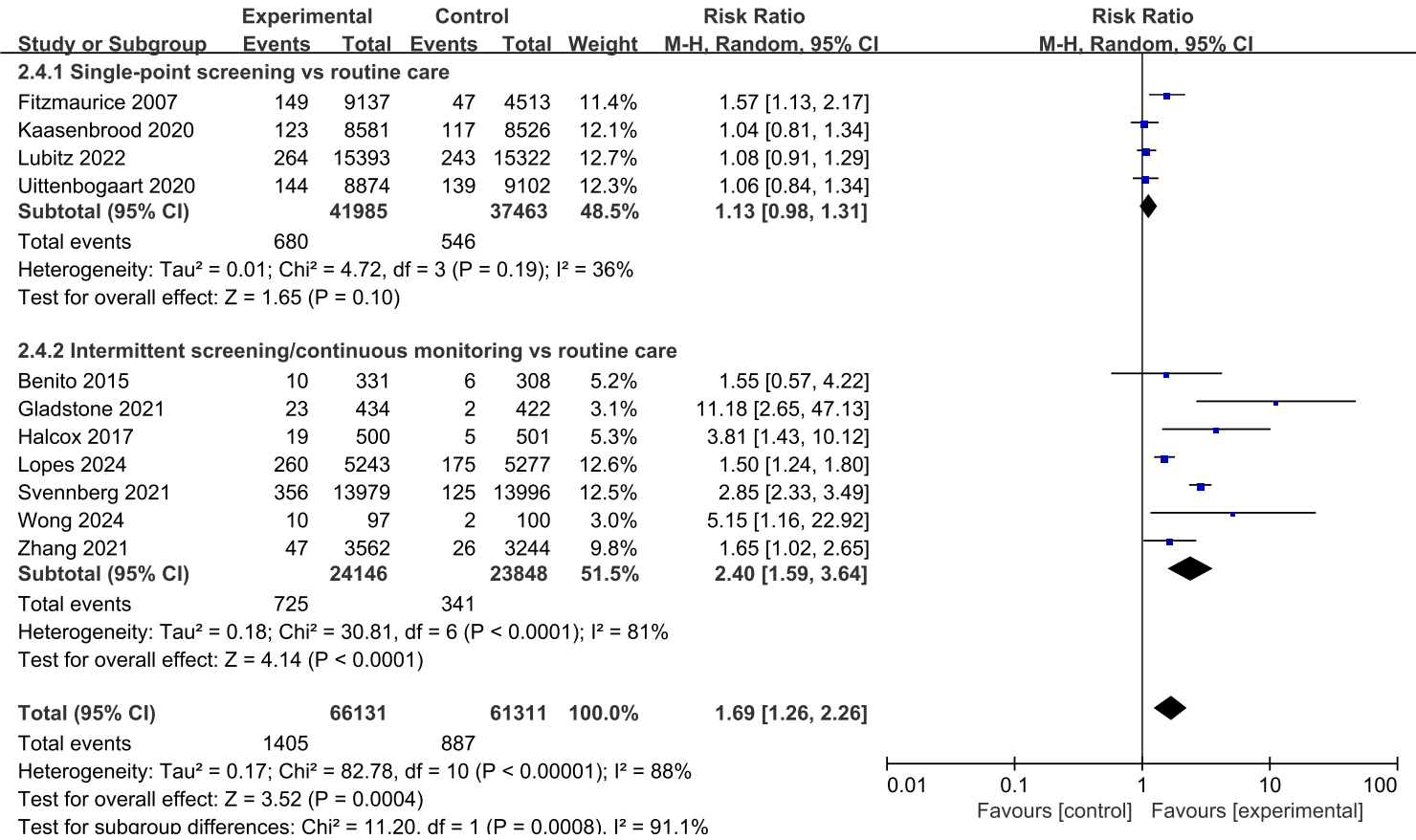

Supplement: Supplementary file 1 [file 2153-8174-26-7-36262-s1.zip › Supplementary Fig 4.pdf]

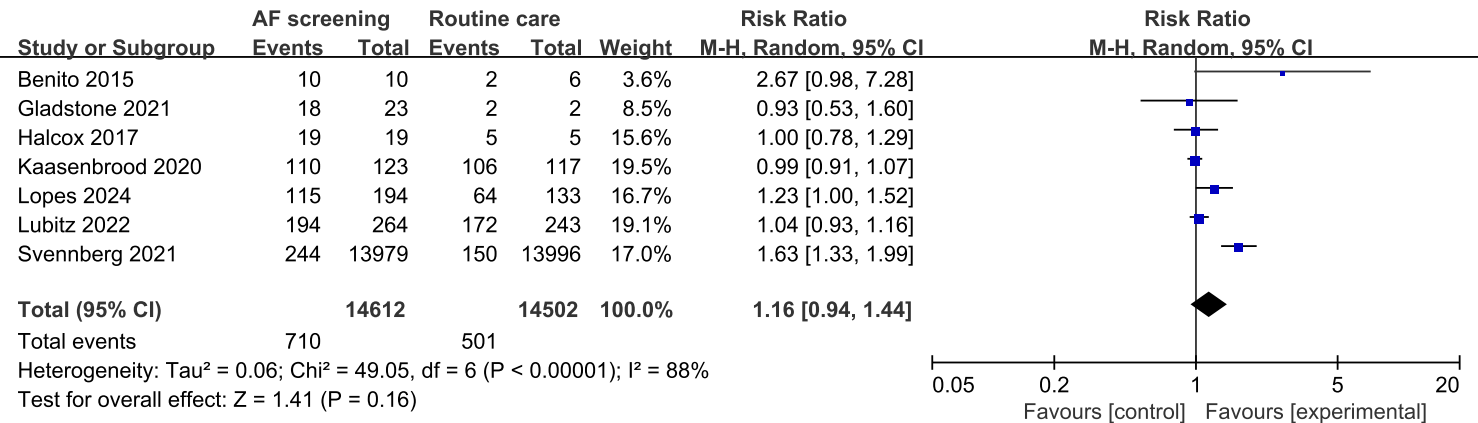

Supplement: Supplementary file 1 [file 2153-8174-26-7-36262-s1.zip › Supplementary Fig 5.pdf]

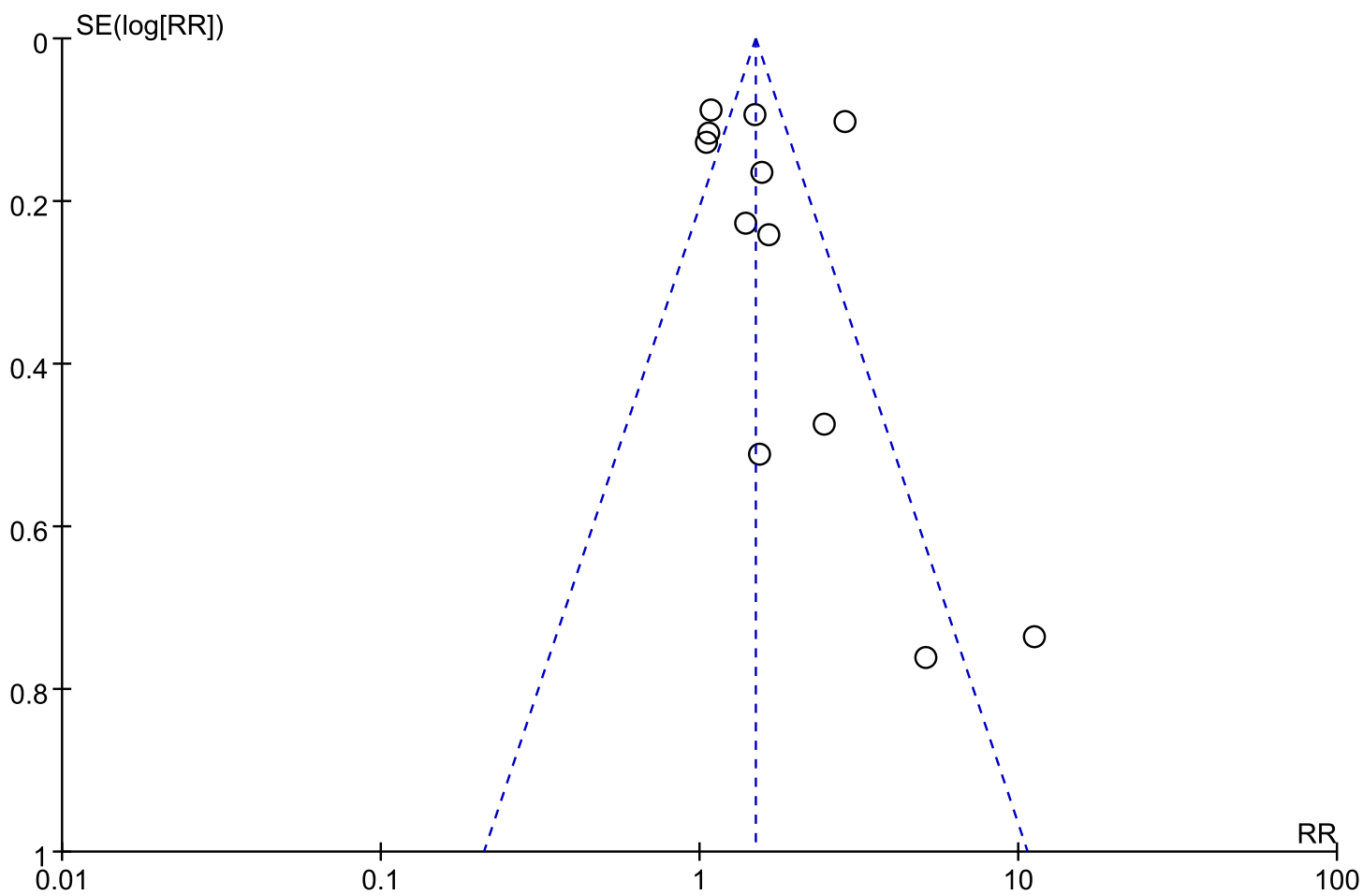

Supplement: Supplementary file 1 [file 2153-8174-26-7-36262-s1.zip › Supplementary Fig 6.pdf]

| Std_Eff | Coef.    | Std. Err. | t    | P> t  | [95% Conf. Interval] |          |
|---------|----------|-----------|------|-------|----------------------|----------|
| slope   | .1878568 | .2241367  | 0.84 | 0.422 | -.311551             | .6872645 |
| bias    | 1.491236 | 1.482489  | 1.01 | 0.338 | -1.811957            | 4.794428 |

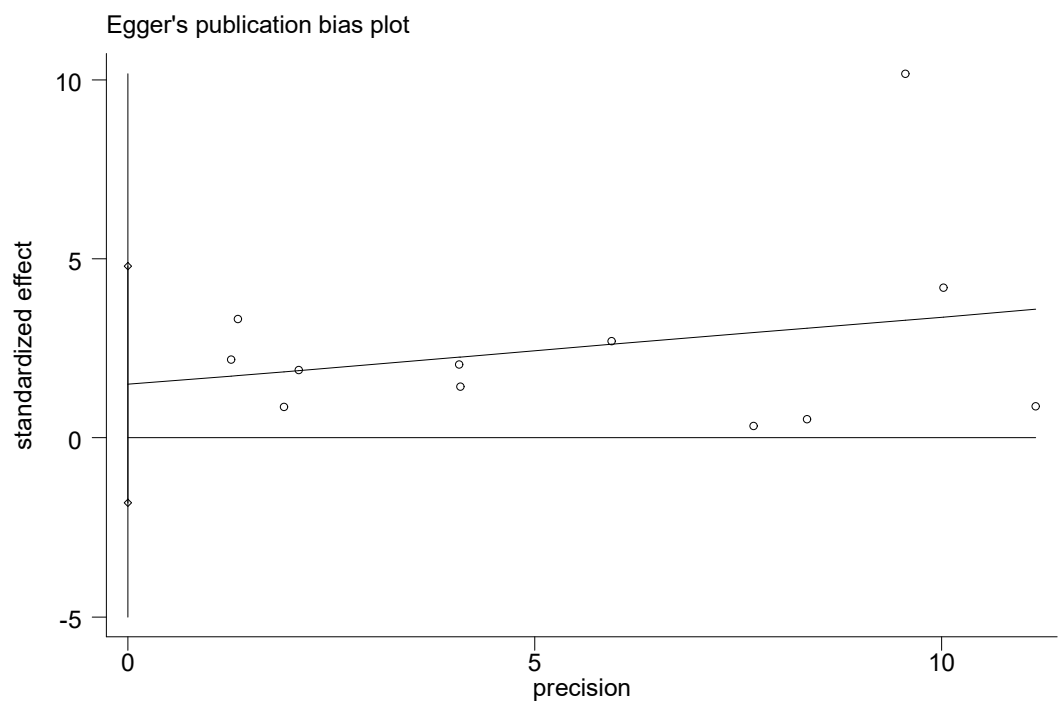

Supplement: Supplementary file 1 [file 2153-8174-26-7-36262-s1.zip › Supplementary Fig 7.pdf]
